# Coronary revascularization outcomes in relation to skilled nursing facility use following hospital discharge

**DOI:** 10.1002/clc.23583

**Published:** 2021-03-23

**Authors:** Samuel T. Savitz, Kristine Falk, Sally C. Stearns, Lexie Grove, Joseph Rossi

**Affiliations:** ^1^ Robert D. and Patricia E. Kern Center for the Science of Health Care Delivery Mayo Clinic Rochester Minnesota USA; ^2^ Division of Health Care Delivery Research Mayo Clinic Rochester Minnesota USA; ^3^ Division of Research Kaiser Permanente Northern California Oakland California USA; ^4^ Department of Health Policy and Management, Gillings School of Global Public Health The University of North Carolina at Chapel Hill Chapel Hill North Carolina USA; ^5^ Division of Cardiology, UNC School of Medicine The University of North Carolina at Chapel Hill Chapel Hill North Carolina USA; ^6^ Cecil G. Sheps Center for Health Services Research The University of North Carolina at Chapel Hill Chapel Hill North Carolina USA

**Keywords:** coronary artery bypass graft, medicare, percutaneous coronary intervention, post‐acute care

## Abstract

**Background:**

Observational analyses comparing coronary artery bypass surgery (CABG) and percutaneous coronary intervention (PCI) among elderly or frail patients are likely biased by treatment selection. PCI is typically chosen for frail patients, while CABG is more common for patients with good recovery potential.

**Hypothesis:**

We hypothesized that skilled nursing facility (SNF) use after revascularization is a measure of relative frailty associated with outcomes following coronary revascularization.

**Methods:**

We used a 20 percent sample of Medicare beneficiaries aged 65 years or older who received inpatient PCI or CABG between 2007–2014. Key explanatory variables were the revascularization strategy and SNF use after revascularization. We used Cox regression to evaluate death and repeat revascularization within one year and logistic regression to evaluate SNF use and 30‐day readmissions/death.

**Results:**

CABG patients were 25.1 percentage points [95% confidence interval: 24.7, 25.5] more likely to use SNF following revascularization than inpatient PCI patients. SNF use was associated with a higher death rate (hazard ratio (HR): 3.19 [3.02, 3.37]) and a 16.2 percentage point (15.5, 16.9) increase in 30‐day readmissions/death. Among patients with SNF use, CABG was associated with a decrease in 30‐day readmissions/death compared to PCI.

**Conclusions:**

While CABG was associated with higher rates of SNF use and 30‐day readmission/death overall, CABG was associated with significantly lower rates of 30‐day readmissions/death among patients with SNF use. The findings suggest that caution is needed in treatment selection for patients at high‐risk for SNF use and that selection of inpatient PCI over CABG may be associated with frailty and worse outcomes for some patients.

## 1 INTRODUCTION

The share of percutaneous coronary intervention (PCI) relative to coronary artery bypass surgery (CABG) that is performed in elderly and frail patients has been growing in recent years.^1^ This pattern is driven in part by dramatic increases in the share of the population that are elderly.^2^, ^3^ PCI, especially in an inpatient setting, may be more suitable for frail patients with limited ability to recovery from major surgery, whereas CABG may be more suitable for patients seeking long‐term durability of revascularization. There has been intense interest in using observational data to compare the effectiveness of these two procedures, but such comparisons have important limitations.

The decision between CABG and PCI involves key tradeoffs that may have different implications for patients who are elderly or frail. CABG is associated with lower rates of mortality in observational research^4^, ^5^, ^6^ and a reduced risk of repeat revascularization in randomized controlled trials (RCTs).^7^, ^8^, ^9^, ^10^, ^11^, ^12^, ^13^ The survival benefits may be more pronounced among elderly patients undergoing revascularization.^6^ The main advantage of PCI is that less time is required for recovery following the procedure,^14^ and it is associated with a lower risk of needing post‐acute care services including skilled nursing facility (SNF) use.^1^ The risk of SNF use among elderly patients is especially important because preoperative frailty is a key predictor for needing SNF care following discharge. While other factors including postoperative complications and prolonged ICU and hospitalization stays are also important predictors,^15^, ^16^ these factors are also associated with greater frailty.^14^, ^17^ Additionally, PCI is associated with a lower risk of stroke.^7^ While some physicians may favor CABG because it is viewed as more durable than PCI and has a reduced risk of repeat revascularization, others physicians may view the reduced risk of morbidity and SNF use associated with PCI as offsetting these advantages. The current guidelines support both treatment alternatives as reasonable options for patients with left main and multivessel coronary artery disease, but do not address the association of likely needing SNF care after discharge with prognosis among patients selected for coronary revascularization.^18^

Despite prior research, gaps exist in our understanding of how the evidence applies to elderly or frail patients. Patients who are very elderly (85 and older) or frail are typically excluded or underrepresented in in randomized trials comparing CABG to PCI.^7^, ^8^, ^9^, ^10^, ^11^, ^12^, ^13^ Additionally, randomized trials do not typically report the use of SNF services after revascularization.^7^, ^8^, ^9^, ^10^, ^11^, ^12^, ^13^ Given the importance of these outcomes for the elderly and frail population, more research in this area would help support selection of patients for treatment.

In this study, we addressed these gaps by using a large data source of elderly and frail patients. We sought to compare the utilization of SNF based on initial revascularization strategy and patient co‐morbidities. We then evaluated the association of treatment and SNF use with key outcomes including all‐cause mortality, repeat revascularization, and 30‐day readmissions or death. In particular, we assessed whether the association of treatment with outcomes was different among patients who did or did not have SNF use after discharge from inpatient revascularization.

## 2 METHODS

We derived the study cohort from a 20 percent random sample of Medicare beneficiaries 65 and older who had simultaneous coverage of Medicare Parts A, B, and D for at least 1 month between 2007–2014. We identified all patients who had PCI or CABG as part of an inpatient admission. We limited our sample to patients who had Medicare fee‐for‐service (Parts A and B) during the month of coronary revascularization since these patients have full claims data. We also excluded patients who had PCI performed as an outpatient procedure because these patients tend to be healthier and not likely to require SNF services. Lastly, we excluded patients who were already in a nursing home before revascularization because such patients were already frail and may be more likely to use SNF after revascularization. All data came from Medicare claims and enrollment records. We received Institutional Review Board Approval from the University of North Carolina at Chapel Hill. We did not obtain informed consent from patients because of the de‐identified nature of this secondary data source.

We focused on three outcomes following live discharge after revascularization: time to all‐cause death, time to first repeat revascularization, and 30‐day readmissions or death. We measured all‐cause death using the Centers for Medicare & Medicaid (CMS) date of death variable. We identified the date of repeat revascularization and readmissions using claims data. The time to all‐cause death and repeat revascularization were measured starting at the discharge date for the index hospitalization during which the patient received an initial revascularization.

The key explanatory variables were whether the patient used Medicare inpatient post‐acute care services within 30 days following discharge and the initial revascularization strategy (PCI or CABG). The inpatient post‐acute care services included stays in long‐term care hospitals (LTAC), inpatient rehabilitation facilities (IRF), and SNF. The vast majority of post‐acute care utilization was for SNF (82.5%) and so we refer to this dichotomous explanatory variable as ‘SNF’ although it also encompasses LTAC and IRF. In addition, we controlled for additional clinical and demographic factors that may be associated with outcomes: age, race/ethnicity, admitting diagnoses, multi‐vessel vs. single‐vessel PCI, the Charlson Comorbidity Index, Medicare/Medicaid dual eligibility, hemodialysis status, and discharge year.

We performed the analyses using Stata version 16 (StataCorp, College Station, TX). We first evaluated SNF use as an intermediate outcome using logistic regression and then included SNF use as an explanatory variable in all subsequent regressions. We did not attempt to adjust for the fact that SNF use technically occurs after revascularization choice, so all estimation results pertain to associations rather than causal effects. We evaluated time‐to‐death and time‐to‐revascularization using Cox proportional hazards regression for deaths and for revascularizations for up to 365 days following discharge. In addition to these regression models, we also evaluated time‐to‐death between 30 and 365 days by excluding patients who died within the first 30 days. We performed this additional regression because SNF use was measured within the first 30 days. Since patients had to survive long enough to receive SNF at some point in the first 30 days, this measure could have led to immortal time bias.^19^ By focusing on patients who survived the first 30 days, we address the possibility of such bias. We evaluated 30‐day readmissions or death using logistic regression.

We evaluated the Cox proportional hazards assumptions using the Schoenfeld residuals test.^20^ We found that the null hypothesis of proportional hazards was rejected for the 0–365 days death model, the 30–365 days death model, and the repeat revascularization model (p < .001). To address the violation of the proportional hazards assumption, we performed sensitivity analyses using extended Cox models. We divided the days into four time periods (0–90 or 30–90 days, 90–180 days, 180–270 days, and 270–365 days). We interacted the key explanatory variables with the time period so that the hazard ratios could vary across the four time periods.

## 3 RESULTS

The descriptive statistics (Table 1) show differences between patients who received inpatient PCI (*N* = 125 077) and patients who received CABG (*N* = 62 334). The use of SNF services in the first 30 days after revascularization was lower for PCI patients (8.2%) than CABG patients (28.4%). PCI patients were also older (75.2 vs. 74.1), and had a slightly higher Charlson Comorbidity Index (2.66 vs. 2.57) compared to CABG patients.

**TABLE 1:** Sample Descriptive Statistics

		Inpatient PCI (*N*=125,077)	CABG (*N*=62,334)
Outcomes			
Death within one year		6,758 (5.4%)	3,082 (4.9%)
Repeat Revascularization within one year		17,888 (14.3%)	3,098 (5.0%)
Number of all‐cause 30‐day readmissions	0	108,723 (86.9%)	51,053 (81.9%)
	1	13,815 (11.0%)	9,482 (15.2%)
	2	2,209 (1.8%)	1,572 (2.5%)
	3+	330 (0.3%)	226 (0.4%)
SNF Use within 30 days		10,246 (8.2%)	17,685 (28.4%)
Control Variables			
Multi‐vessel PCI		23,549 (18.8%)	NA
Mean Age (SD)		75.24 (7.06)	74.12 (6.05)
Age Group	65‐69	36,154 (28.9%)	19,108 (30.7%)
	70‐74	30,425 (24.3%)	17,138 (27.5%)
	75‐79	25,460 (20.4%)	14,324 (23.0%)
	80‐84	19,544 (15.6%)	8,904 (14.3%)
	85+	13,494 (10.8%)	2,860 (4.6%)
	White	104,335 (83.4%)	53,352 (85.6%)
Race Group	Black	8,544 (6.8%)	3,223 (5.2%)
	Hispanic	7,785 (6.2%)	3,725 (6.0%)
	Other	4,413 (3.5%)	2,034 (3.3%)
Male		66,036 (52.8%)	40,068 (64.3%)
ICD9 Admitting Diagnosis	STEMI	20,759 (20.4%)	3,259 (13.8%)
	NSTEMI	27,339 (26.9%)	7,206 (30.6%)
	Unstable Angina	20,335 (20.0%)	5,016 (21.3%)
	Stable CAD	31,573 (31.1%)	7,918 (33.6%)
	Other	1,512 (1.5%)	160 (0.7%)
Average Charlson	Mean (SD)	2.66 (1.97)	2.57 (1.90)
Hemodialysis		1,258 (1.0%)	1,127 (1.8%)
Congestive Heart Failure		38,157 (30.5%)	21,392 (34.3%)
Peripheral Vascular Disease		23,835 (19.1%)	12,728 (20.4%)
Cerebrovascular Disease		15,662 (12.5%)	10,984 (17.6%)
Chronic Obstructive Pulmonary Disease		35,700 (28.5%)	18,141 (29.1%)
Dementia		3,069 (2.5%)	767 (1.2%)
Paralysis		957 (0.8%)	677 (1.1%)
Diabetes Mellitus		51,093 (40.8%)	26,982 (43.3%)
Diabetes Mellitus with Complications		16,893 (13.5%)	8,481 (13.6%)
Renal Disease		22,897 (18.3%)	10,849 (17.4%)
Mild Liver Disease		832 (0.7%)	441 (0.7%)
Moderate‐Severe Liver Disease		316 (0.3%)	180 (0.3%)
Ulcers		2,013 (1.6%)	929 (1.5%)
Rheumatologic Disease		4,792 (3.8%)	1,865 (3.0%)
Cancer		12,590 (10.1%)	6,024 (9.7%)
Metastatic Cancer		143 (0.1%)	72 (0.1%)

**FIGURE 1:**
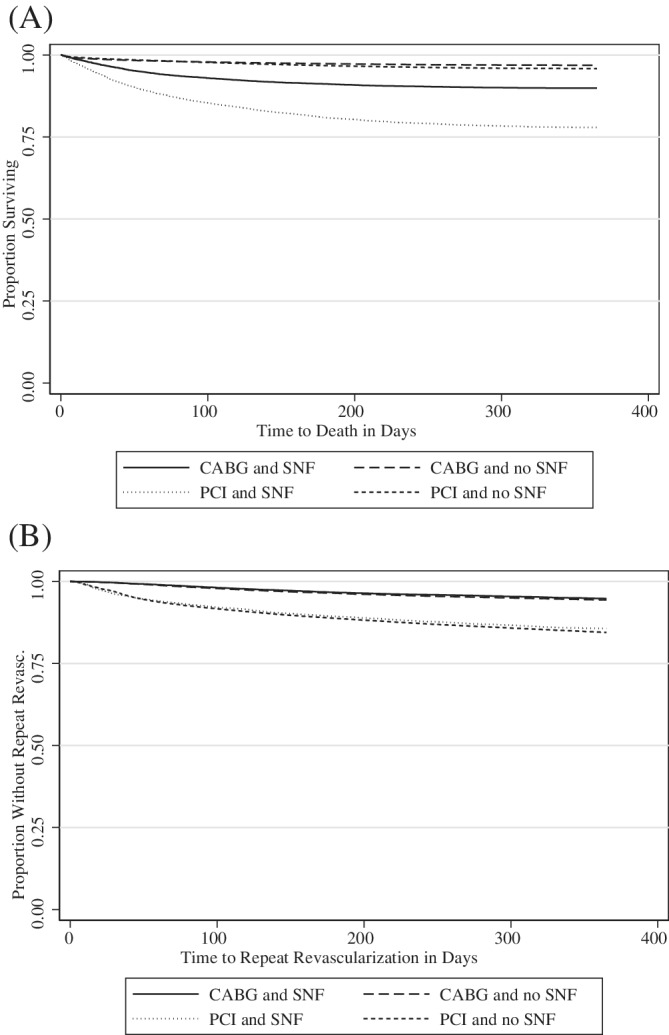
(A) Kaplan–Meier Curve for Time‐to‐death. (B) Kaplan–Meier Curve for Time to Repeat Revascularization. CABG, coronary artery bypass grafting; SNF, skilled nursing facility; PCI, percutaneous coronary intervention

The unadjusted outcomes are stratified by treatment (PCI and CABG), age group, and SNF use (Table 2). The percentage of patients with SNF use increased with age. Among CABG patients, the percentage with SNF use was 17.5% in the 65–69 age group and 56.5% in the 85+ age group. The pattern was similar for PCI patients, although the respective percentages were lower than for CABG patients; the percentage of patients with SNF use was 3.8% in the 65–69 age group and 20.5% in the 85+ age group. The average Charlson score was higher for patients with versus without SNF use both for CABG (3.13 vs. 2.34) and PCI (3.93 vs. 2.55). CABG patients with SNF use had worse outcomes relative to CABG patients without SNF use in terms of death (9.8% vs. 3.0%) and 30‐day readmissions/death (29.8% vs. 15.1%). The differences were even larger for PCI patients with SNF use compared to PCI patients without SNF use for death (21.2% vs. 4.0%) and 30‐day readmission/death (39.9% vs. 11.7%). The Kaplan–Meier curve for death (Figure 1(A)) illustrates the wide gap in time‐to‐death between patients with and without SNF use for both CABG and PCI. The Kaplan–Meier curves for repeat revascularization (Figure 1(B)) show that the incidence of repeat revascularization was similar for CABG patients regardless of SNF use. The incidence of repeat revascularization was higher for PCI patients compared to CABG patients, but the incidence was similar for patients with and without SNF use.

**TABLE 2:** Descriptive statistics stratified by SNF use

Treatment	Age	Total Patients	Patients Without SNF Stay Within 30 Days of Discharge					Patients With SNF Stay Within 30 Days of Discharge				
			Patients (% of patients without SNF use)	Average Charlson Score	% Died within 1 year	% Repeat Revascularization within 1 year	% Read mission or death within 30 days	Patients (% of patients with SNF use)	Average Charlson Score	% Died within 1 year	% Repeat Revascularization within 1 year	% Read mission or death within 30 days
CABG	65‐69	19,108	15,767 (82.5%)	2.24 (0.01)	2.00% (0.11%)	6.15% (0.19%)	13.36% (0.27%)	3,341 (17.5%)	3.35 (0.04)	8.23% (0.48%)	5.81% (0.4%)	31.1% (0.8%)
	70‐74	17,138	13,146 (76.7%)	2.33 (0.02)	2.66% (0.14%)	5.23% (0.19%)	14.72% (0.31%)	3,992 (23.3%)	3.30 (0.03)	9.49% (0.46%)	5.46% (0.36%)	29.58% (0.72%)
	75‐79	14,324	9,652 (67.4%)	2.42 (0.02)	3.50% (0.19%)	4.78% (0.22%)	15.84% (0.37%)	4,672 (32.6%)	3.09 (0.03)	10.12% (0.44%)	4.35% (0.3%)	29.69% (0.67%)
	80‐84	8,904	4,840 (54.4%)	2.48 (0.03)	5.06% (0.32%)	3.93% (0.28%)	18.45% (0.56%)	4,064 (45.6%)	2.93 (0.03)	10.68% (0.48%)	3.52% (0.29%)	29.48% (0.72%)
	85+	2,860	1,244 (43.5%)	2.65 (0.05)	8.04% (0.77%)	2.81% (0.47%)	22.43% (1.18%)	1,616 (56.5%)	2.91 (0.05)	10.64% (0.77%)	3.03% (0.43%)	28.65% (1.13%)
	Total	62,334	44,649 (71.6%)	2.34 (0.01)	3.02% (0.08%)	5.25% (0.11%)	15.10% (0.17%)	17,685 (28.4%)	3.13 (0.01)	9.80% (0.22%)	4.56% (0.16%)	29.79% (0.34%)
Inpatient PCI	65‐69	36,154	34,772 (96.2%)	2.37 (0.01)	2.67% (0.09%)	16.01% (0.2%)	10.05% (0.16%)	1,382 (3.8%)	4.24 (0.06)	18.60% (1.05%)	17.44% (1.02%)	40.30% (1.32%)
	70‐74	30,425	28,739 (94.5%)	2.47 (0.01)	3.30% (0.11%)	15.02% (0.21%)	10.74% (0.18%)	1,686 (5.5%)	4.08 (0.05)	20.34% (0.98%)	13.05% (0.82%)	40.81% (1.20%)
	75‐79	25,460	23,381 (91.8%)	2.63 (0.01)	4.18% (0.13%)	14.65% (0.23%)	12.21% (0.21%)	2,079 (8.2%)	4.09 (0.05)	21.65% (0.9%)	11.45% (0.7%)	40.74% (1.08%)
	80‐84	19,544	17,218 (88.1%)	2.75 (0.01)	5.06% (0.17%)	13.54% (0.26%)	13.30% (0.26%)	2,326 (11.9%)	3.83 (0.04)	20.55% (0.84%)	11.95% (0.67%)	39.94% (1.02%)
	85+	13,494	10,721 (79.5%)	2.85 (0.02)	8.04% (0.26%)	11.63% (0.31%)	15.86% (0.35%)	2,773 (20.5%)	3.63 (0.04)	23.15% (0.8%)	8.73% (0.54%)	38.30% (0.92%)
	Total	125,077	114,831 (91.8%)	2.55 (0.01)	4.00% (0.06%)	14.71% (0.1%)	11.69% (0.09%)	10,246 (8.2%)	3.93 (0.02)	21.18% (0.4%)	11.90% (0.32%)	39.85% (0.48%)

The adjusted outcomes were similar to the unadjusted outcomes (Table 3). For the model predicting SNF use, CABG was associated with a 25.1 percentage point (95% confidence interval: 24.7, 25.5) increase in the predicted percentage of patients with SNF use after revascularization. Advanced age was also a strong predictor, with patients 85 and older being 20.5 percentage points (19.8, 21.2) more likely to have SNF use after revascularization.

**TABLE 3:** Regression results

	Logit Model SNF Use within 30 Days	Cox Model Death 0‐365 Days	Cox Model Death 30‐365 Days	Cox Model Revasculariztion	Logit Model Readmission/ Death
	Marginal Effect (Standard Error)	Marginal Effect (Standard Error)	Hazard Ratio (Standard Error)	Hazard Ratio (Standard Error)	Hazard Ratio (Standard Error)
PCI Inpatient Single Vessel	Reference				
PCI Multi Vessel	1.14%***	1.07*	1.01	1.14***	0.38%
	(0.57,1.71)	(1.00,1.13)	(0.94,1.08)	(1.10,1.18)	(‐0.14,0.91)
CABG	25.07%***	0.85***	0.63***	0.35***	3.46%***
	(24.65,25.50)	(0.80,0.91)	(0.58,0.69)	(0.34,0.37)	(3.04,3.87)
SNF Use within 30 Days	NA	3.19***	3.42***	0.89***	16.18%***
		(3.02,3.37)	(3.21,3.66)	(0.84,0.94)	(15.52,16.85)
SNF Use x CABG Interaction	NA	0.72***	0.85**	1.01	NA
		(0.66,0.79)	(0.76,0.95)	(0.92,1.12)	
Age 65‐69	Reference				
Age 70‐74	2.95%***	1.25***	1.22***	0.92***	0.79%***
	(2.60,3.30)	(1.18,1.34)	(1.13,1.31)	(0.89,0.95)	(0.36,1.21)
Age 75‐79	7.30%***	1.56***	1.48***	0.87***	1.88%***
	(6.90,7.70)	(1.46,1.66)	(1.38,1.60)	(0.84,0.91)	(1.42,2.33)
Age 80‐84	13.16%***	1.82***	1.79***	0.81***	2.82%***
	(12.67,13.65)	(1.70,1.94)	(1.65,1.93)	(0.77,0.84)	(2.31,3.33)
Age 85+	20.50%***	2.45***	2.33***	0.70***	4.27%***
	(19.82,21.18)	(2.29,2.62)	(2.14,2.53)	(0.66,0.74)	(3.63,4.91)
White	Reference				
Black	‐0.07%	1.06	1.19***	1.05	‐0.14%
	(‐0.69,0.54)	(0.98,1.14)	(1.09,1.30)	(1.00,1.11)	(‐0.78,0.49)
Other	‐3.10%***	0.82**	0.86*	1.08*	‐1.17%**
	(‐3.85,‐2.35)	(0.73,0.93)	(0.74,0.99)	(1.00,1.16)	(‐2.03,‐0.32)
Hispanic	‐3.33%***	0.93	0.96	1.14***	‐0.30%
	(‐3.89,‐2.77)	(0.86,1.01)	(0.87,1.07)	(1.08,1.20)	(‐0.95,0.36)
Male	‐6.05%***	1.13***	1.17***	1.05***	‐2.13%***
	(‐6.36,‐5.74)	(1.08,1.17)	(1.11,1.23)	(1.02,1.08)	(‐2.46,‐1.81)
Hemodialysis	12.52%***	3.36***	2.98***	1.96***	31.50%***
	(11.06,13.98)	(3.11,3.63)	(2.70,3.29)	(1.78,2.17)	(29.43,33.57)
Charlson	2.30%***	1.26***	1.28***	1.09***	2.11%***
	(2.23,2.37)	(1.25,1.27)	(1.26,1.29)	(1.08,1.09)	(2.03,2.19)
STEMI	Reference				
NSTEMI	‐2.55%***	0.77***	0.92*	0.86***	‐1.62%***
	(‐3.12,‐1.97)	(0.73,0.82)	(0.85,0.99)	(0.82,0.90)	(‐2.16,‐1.07)
Unstable Angina	‐8.52%***	0.56***	0.75***	0.51***	‐2.04%***
	(‐9.10,‐7.93)	(0.52,0.60)	(0.68,0.82)	(0.45,0.58)	(‐2.63,‐1.46)
Stable CAD	‐7.37%***	0.70***	0.95	0.89***	‐2.32%***
	(‐7.92,‐6.83)	(0.66,0.74)	(0.88,1.03)	(0.85,0.92)	(‐2.84,‐1.79)
Other	‐4.37%***	1.58***	1.80***	0.93**	1.46%**
	(‐5.28,‐3.46)	(1.44,1.74)	(1.60,2.04)	(0.89,0.97)	(0.40,2.53)
Full Dual Eligible	5.34%***	1.02	1.10	1.02	1.66%***
	(4.61,6.06)	(0.95,1.11)	(1.00,1.20)	(0.97,1.07)	(0.99,2.33)
Partial Dual Eligible	2.01%***	1.17**	1.12	1.01	2.11%***
	(1.06,2.95)	(1.04,1.30)	(0.98,1.29)	(0.93,1.08)	(1.16,3.07)
Observations	187,411	187,411	181,999	187,411	187,411
C‐Statistic	NA	0.7745	0.7782	0.6435	NA
Pseudo‐R2	0.1869	NA	NA	NA	0.0806

*Note:* Regressions also controlled for year fixed effects. *** *p* < 0.001, ** *p* < 0.01, * *p* < 0.05.

For all‐cause mortality, SNF use was associated with a much greater rate of death for the 0–365 days death model (hazard ratio (HR): 3.19 [95% confidence interval: 3.02, 3.37]). The rate of death was also lower for patients who received CABG compared to single‐vessel inpatient PCI (0.85 [0.80, 0.91]). However, the rate of death was slightly for patients with multi‐vessel PCI compared to patients with single‐vessel PCI (1.07 [1.00, 1.13]). Overall, the results for the 30–365 days death model were similar in magnitude and direction as the 0–365 days model. However, the difference for CABG compared to inpatient PCI did become larger (0.63 [0.58,0.69]) and the difference for multi‐vessel PCI compared to single‐vessel PCI became non‐significant.

For repeat revascularization, SNF use was associated with a slightly lower rate (0.89 [0.84, 0.94]). The rate of repeat revascularization was much lower for patients who received CABG compared to inpatient PCI (0.35 [0.34, 0.37]) and slightly higher for patients with multi‐vessel PCI (1.14 [1.10, 1.18]).

**FIGURE 2:**
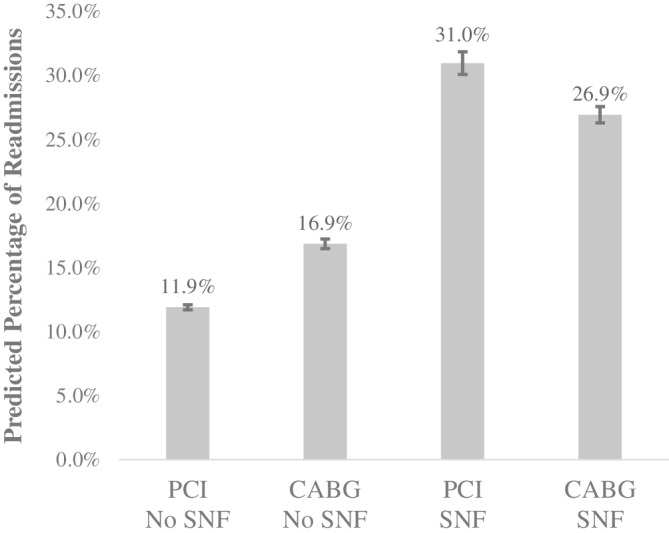
Predicted Percentage of Readmission by Revascularization Type and SNF Use. CABG, coronary artery bypass grafting; SNF, skilled nursing facility; PCI, percutaneous coronary intervention

SNF use was associated with a higher predicted percentage of 30‐day readmissions/death (Table 3). The marginal effect for SNF use was a 16.2 percentage point (15.5, 16.9) increase in the predicted percentage of patients readmitted/dead. The strength of the association of SNF use with readmissions/death differed between patients who received CABG and PCI (Figure 2). The predicted percentage of readmissions/death was higher for CABG patients (16.9% [16.5%, 17.2%]) compared to PCI patients (11.9% [11.7%, 12.1%]) among those without SNF use. However, the relative predicted percentages of readmissions/death reversed and were significantly lower for CABG patients (26.9% [26.3%, 27.6%] compared to PCI patients (31.0% [30.1%, 31.9%] with SNF use.

The extended Cox model results allowed the HRs for death to vary by time period (Supplemental Figure 1A). The HRs for PCI and CABG patients with SNF compared to PCI patients without SNF were greater in magnitude for days 0–90 and the magnitude steadily declined to the period for days 270–365. The findings were similar for the 30–365 days model (Supplemental Figure 1B). This pattern suggests that the association between SNF use and death was stronger for time periods closer to the date of revascularization.

For repeat revascularization, the results were more stable over the time periods (Supplemental Figure 1C). The rate of repeat revascularization for PCI with SNF use was slightly lower and the rate for CABG with SNF was much lower compared to PCI without SNF across all four periods.

## 4 DISCUSSION

In a large sample of patients receiving inpatient coronary revascularization, SNF use was more common following CABG compared to PCI. However, among patients with SNF utilization, mortality was significantly higher among patients selected for PCI. These findings were present despite adjustment for age, sex, multi‐vessel vs. single‐vessel PCI and medical comorbidities, suggesting that unmeasured confounders are associated with SNF use and subsequent mortality, rather than revascularization itself. In addition, SNF use overall was associated with lower rates of revascularization despite higher mortality, suggesting that patients transferred to SNF were less likely to be offered repeat procedures and were likely treated more conservatively due to higher burden of comorbidities.

We found that SNF use was strongly associated with an increased all‐cause mortality and 30‐day readmissions. While it is not surprising that patients with SNF use had worse outcomes since these patients are more likely to be in worse health, have experienced postoperative complications and have had prolonged stays, this association was significant despite controlling for comorbidity burden and age. The strong association with all‐cause mortality also remained after excluding patients who died within the first 30 days (the same period when SNF use was measured). We also found that the association was strongest in the first period after revascularization and weaker in later periods, suggesting that post‐discharge SNF utilization is a particularly strong predictor of early mortality.

These results add important context to the tradeoffs that exist when selecting a revascularization strategy for elderly and frail patients. Like previous studies, we found that patients who received CABG had a slightly lower all‐cause mortality^4^, ^5^, ^6^ and a lower rate of repeat revascularization compared to patients who received inpatient PCI.^7^, ^8^, ^9^, ^10^, ^11^, ^12^, ^13^ We also confirmed that patients who received CABG were more likely to have SNF use.^1^ This finding is due at least in part to the higher need for recovery and SNF care following a more invasive procedure. While this result was expected, the magnitude of the increase in SNF use for CABG compared to inpatient PCI has not been well‐quantified in previous research and is not typically reported in randomized clinical trials as an important clinical endpoint. In contrast to prior research,^21^, ^22^ we found that CABG was associated with a higher risk of 30‐day readmissions or death compared to inpatient PCI. However, the prior studies focused on much longer timeframes than 30‐days.^21^, ^22^ Taken together, these findings suggest that the choice between CABG and inpatient PCI depends on the relative value of the slight increase in longevity and large decrease in repeat revascularization for CABG weighed against the decrease in short‐term readmissions or death, SNF use, and recovery time for PCI.

SNF use was a stronger predictor for poor outcomes among patients who received inpatient PCI compared CABG. For this subset, CABG was associated with a decrease in mortality and the percentage of patients with readmission or mortality compared to PCI. However, the proportion of CABG patients who required SNF use was much higher. Additionally, PCI inpatients with SNF use also had a higher comorbidity burden and were older than CABG patients with SNF use. These results suggest that the small subset of patients who received PCI and required SNF use were a group that was at especially high risk for poor outcomes. This is particularly important given recent findings of randomized clinical trials demonstrating the effectiveness of medical therapy alone as an initial treatment strategy for patients with stable coronary artery disease.^23^, ^24^ For patients with advanced frailty likely to require SNF utilization and associated poor prognosis, clinicians may be more likely to consider an initial trial of medical therapy alone rather than invasive evaluation.

These findings also have important implications with respect to treatment selection for revascularization patients who are elderly and frail who remain candidates for invasive evaluation. First, the findings support the concerns about the risk of CABG use in this frail and elderly population. CABG patients were much more likely to require SNF use despite being younger and having a lower average comorbidity burden. The results suggest greater caution may be warranted when selecting treatment for patients at high risk for SNF use. Second, the results suggest that there is selection in favor of PCI among patients with extreme frailty and more advanced age at baseline. Inpatient PCI patients were older, had a higher comorbidity burden, and had a worse prognosis conditional on having SNF use. This selection of patients with extreme frailty to receive inpatient PCI may be reasonable since PCI is a minimally invasive procedure whereas CABG is an invasive surgery that was associated with a greater risk of post‐revascularization SNF use. However, the poor outcomes among the subset of inpatients with PCI and SNF use may reflect a need for greater consideration for whether patients with extreme frailty are good candidates for revascularization. Additionally, such selection presents a challenge for observational research that compares outcomes between PCI and CABG. The patients who receive inpatient PCI may be more frail in unobservable ways and have a worse prognosis prior to revascularization. This issue may partially explain the survival advantage for CABG compared to PCI that was found in this study and in prior observational research.^4^, ^5^, ^6^ Third, current results from clinical trials are unlikely to determine the optimal revascularization strategy for elderly and frail patients. Physicians are unlikely to allow this complex treatment selection to be left to chance, and improved ability to analyze observational data will remain an important method of evaluating the comparative effectiveness of revascularization strategies.

The strengths of this cohort include the large sample size and that it is nationally representative of elderly Medicare fee‐for‐service beneficiaries. The study also has several limitations. First, we excluded Medicare Advantage beneficiaries and our results may not generalize to these patients. Second, as an observational study, the associations between receipt of CABG or inpatient PCI and outcomes should not be interpreted as causal. A randomized controlled trial would be necessary to evaluate the comparative effectiveness of inpatient PCI and CABG in this population. Third, SNF use is an indicator associated with functional status, postoperative complications, and prolong stays that occurs after revascularization and did not cause the observed associations with outcomes. Fourth, while we were able to control for comorbidity burden and admission diagnoses, we were unable to control for clinical fa//ctors that are not available in claims data such as severity of CAD or anatomic variants.^25^

In summary, among a large cohort of elderly and frail Medicare fee‐for‐service beneficiaries receiving revascularization, we found that CABG was associated with a lower rate of death and repeat revascularization, but a higher risk of readmissions. We also found that SNF use was more likely among CABG patients and that SNF use was associated with poor outcomes, particularly among patients receiving inpatient PCI. These observations highlight the important limitations in conducting observational research in the comparative effectiveness of CABG compared to inpatient PCI. The results suggest that caution is needed in treatment selection for patients at high‐risk for SNF use and that selection of inpatient PCI over CABG may be associated with greater frailty and a worse overall prognosis.

## CONFLICT OF INTEREST

None.

## Supporting information


**Figure S1** XXX

## Data Availability

The data that support the findings of this study are available from the Centers for Medicare & Medicaid Services. Restrictions apply to the availability of these data, which were used under a Data Use Agreement (DUA) for this study. Data are available by submitting a DUA application (https://www.resdac.org/request-materials/rif-data-use-agreement-dua) to ResDAC.
