# Supplementary figures and images for: Coronary revascularization outcomes in relation to skilled nursing facility use following hospital discharge
Source: Clin Cardiol. 2021 Mar 23;44(5):627–35. doi: 10.1002/clc.23583 (PMC8119835; doi:10.1002/clc.23583)

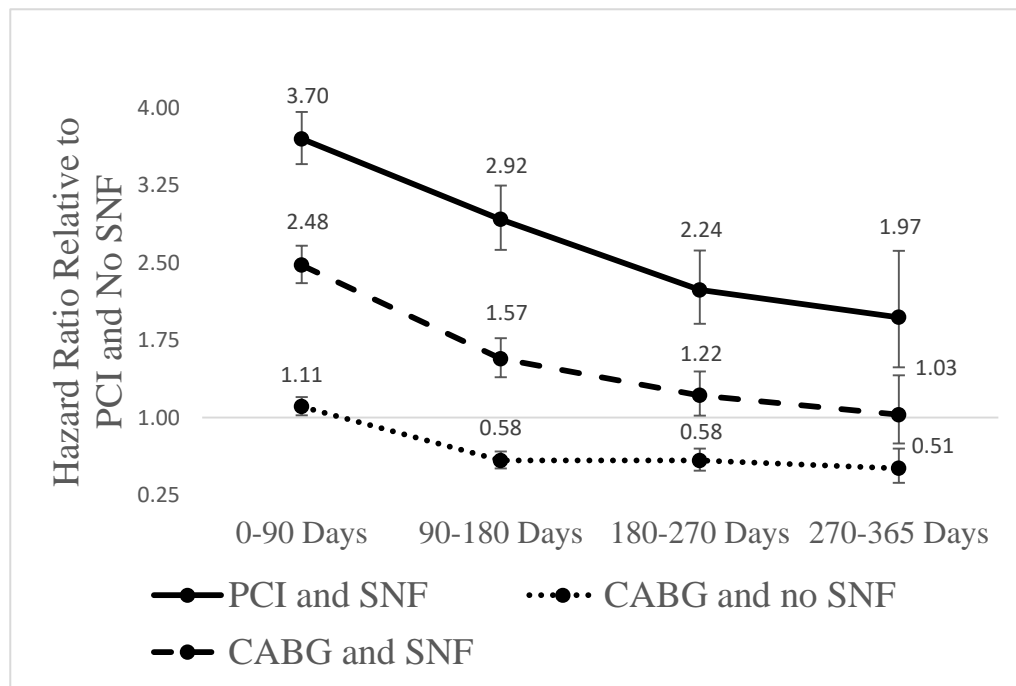

Supplement: Supplementary file 1 — Figure S1 XXX [file CLC-44-627-s001.zip › CLC_23583_Supplemental Figure 1A.pdf]

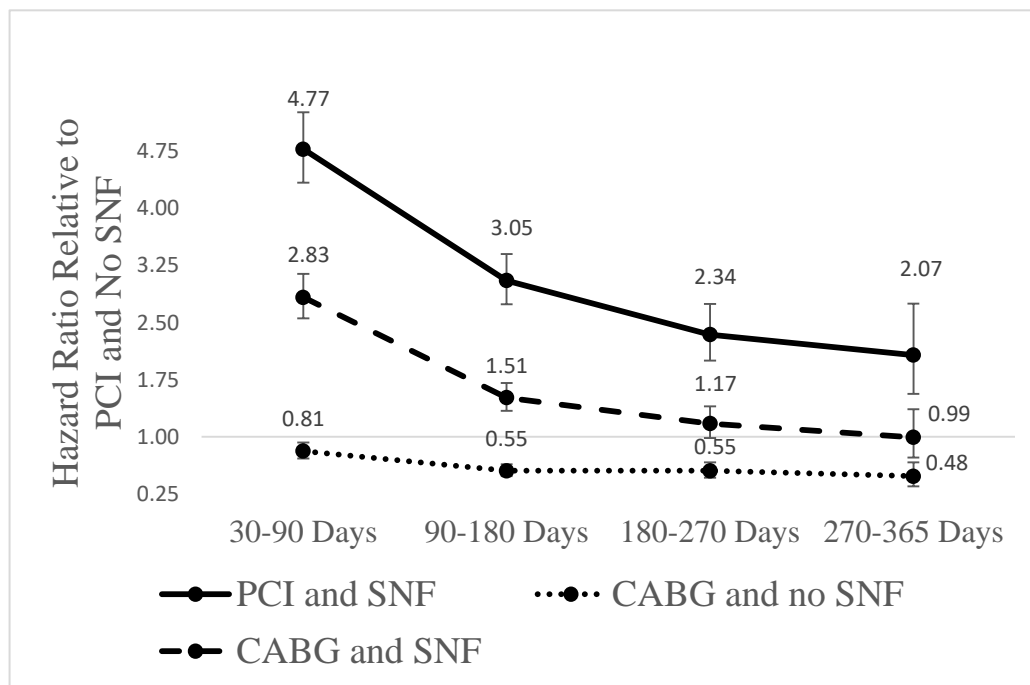

Supplement: Supplementary file 1 — Figure S1 XXX [file CLC-44-627-s001.zip › CLC_23583_Supplemental Figure 1B.pdf]

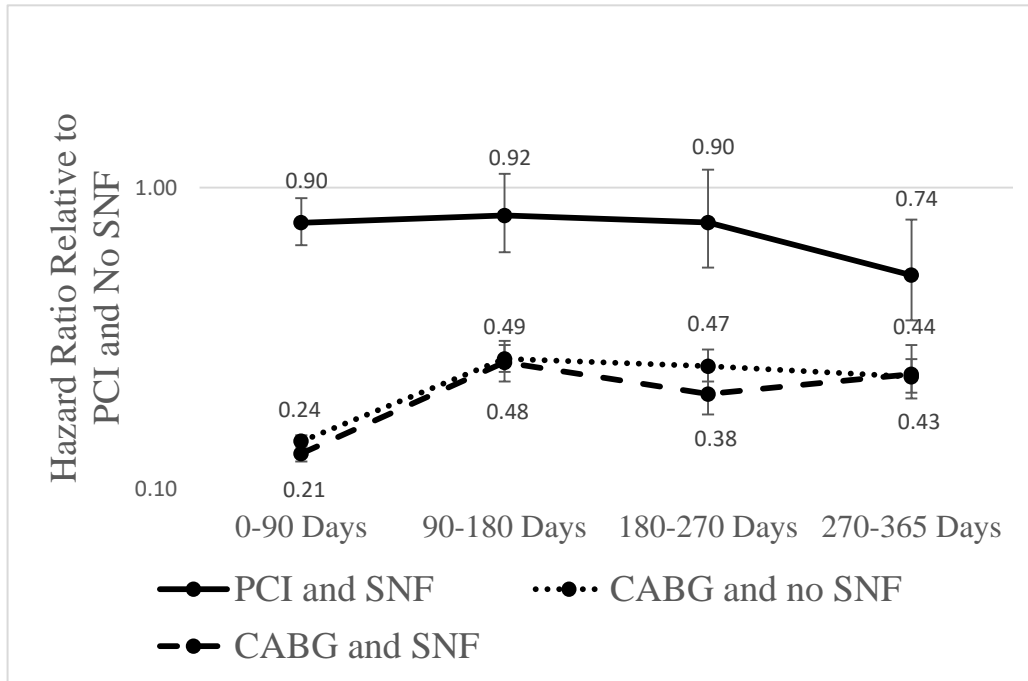

Supplement: Supplementary file 1 — Figure S1 XXX [file CLC-44-627-s001.zip › CLC_23583_Supplemental Figure 1C.pdf]
